# Macrophage polarization-associated lnc-Ma301 interacts with caprin-1 to inhibit hepatocellular carcinoma metastasis through the Akt/Erk1 pathway

**DOI:** 10.1186/s12935-021-02133-1

**Published:** 2021-08-10

**Authors:** Hong-Lin Luo, Tao Luo, Jun-Jie Liu, Fei-Xiang Wu, Tao Bai, Chao Ou, Jie Chen, Le-Qun Li, Jian-Hong Zhong

**Affiliations:** 1 Department of Hepatobiliary Surgery, Guangxi Medical University Cancer Hospital, He Di Rd 71, Nanning, 530021 People’s Republic of China; 2 Key Laboratory of High-Incidence Tumor Early Prevention and Treatment, Ministry of Education, Nanning, 530021 People’s Republic of China; 3 Department of Ultrasound, Guangxi Medical University Cancer Hospital, Nanning, 530021 People’s Republic of China; 4 Department of Clinical Laboratory Medicine, Guangxi Medical University Cancer Hospital, Nanning, 530021 People’s Republic of China

**Keywords:** Hepatocellular carcinoma, Long non-coding RNAs, Cancer migration, Epithelial–mesenchymal transition, Metastasis

## Abstract

**Background:**

Epithelial–mesenchymal transition (EMT) promotes migration, invasion, and metastasis of hepatocellular carcinoma (HCC) cells. The molecular mechanisms behind EMT and metastasis in HCC remain unclear.

**Methods:**

Microarray analysis was used to identify lncRNAs expression during polarization of U937 macrophages from M2 to M1 phenotype. The expression of the identified lncRNA was compared between clinical samples of HCC tissues or adjacent normal tissues, as well as between HCC and normal liver cell lines. lnc-Ma301 was overexpressed or knocked-down in HCC cell lines, and the effects were assessed in vitro and in vivo. Interactions among lnc-Ma301 and its potential downstream targets caprin-1 were investigated in HCC cell lines. Effects of lnc-Ma301 over- and underexpression on the Akt/Erk1 signaling pathways were examined.

**Results:**

Microarray analyses identified lnc-Ma301 as one of the most overexpressed long non-coding RNAs during polarization of U937 macrophages from M2 to M1 phenotype. Lnc-Ma301 showed lower expression in HCC tissues than in adjacent normal tissues, and lower expression was associated with worse prognosis. Activation of lnc-Ma301 inhibited cell proliferation, migration and EMT in HCC cell cultures, and it inhibited lung metastasis of HCC tumors in mice. Mechanistic studies suggested that lnc-Ma301 interacts with caprin-1 to inhibit HCC metastasis and EMT through Akt/Erk1 pathway.

**Conclusions:**

Lnc-Ma301 may help regulate onset and metastasis of HCC.

**Supplementary Information:**

The online version contains supplementary material available at 10.1186/s12935-021-02133-1.

## Introduction

The global incidence of hepatocellular carcinoma (HCC) is rising worldwide [1]. Due to the lack of significant symptoms and signs at an early stage of the disease, more than 50% of patients with HCC are first diagnosed at an advanced tumor stage [2]. Moreover, lung metastasis occurs in many HCC patients with advanced disease [3]. These two factors make long-term prognosis poor for HCC patients [4]. The concrete mechanisms of HCC pathogenesis are not fully understood [3]. Epithelial–mesenchymal transition (EMT) is known to promote HCC cell migration, invasion, and metastasis. EMT is also involved in the cascade of signaling events that induce HCC metastasis [5, 6]. However, the molecular details about how EMT is induced in HCC and how it may drive metastasis remain unclear.

Macrophages can exhibit an M1 phenotype (“classically activated”) or M2 phenotype (“alternatively activated”) [7]. M1 macrophages can inhibit tumor cell aggregation, while M2 macrophages can facilitate tumor angiogenesis [8]. Tumor-associated macrophages (TAMs) mainly present the M2 phenotype and have important roles in both tumor progression and therapeutic response (including HCC) [9–11]. Moreover, the function and polarization of epigenetic modification-mediated TAM linked to the pathogenesis of HCC [11].

Long non-coding RNAs (lncRNAs) regulate gene expression. Several lncRNAs have been found to contribute to tumorigenesis and progression of HCC by binding to proteins, RNA, and DNA or by encoding small peptides [12–14]. In particular, interactions between lncRNAs and RNA-binding proteins have been well studied [15, 16]. Some lncRNAs promote HCC tumor growth and metastasis by promoting polarization of macrophages to the M2 phenotype [17, 18]. Considering the essential roles of macrophages and lncRNAs in the tumorigenesis and progression of HCC, it is important to determine the expression of lncRNAs in macrophages during their polarization from the M2 to the M1 phenotype. Drugs targeted to such lncRNAs on both directions of the M1–M2 axis may prevent the tumorigenesis and progression of HCC.

In the present study, we used microarray analyses to analyze the expression of lncRNAs and mRNAs in the process of M2 to M1 macrophage polarization in human monocytic U937 cells [19]. We found that lnc-Ma301 was one the most highly expressed lncRNAs in M1 macrophages. Microarray analyses revealed that lnc-Ma301 regulates epidermal growth factor receptor substrate 15, which is related to HCC invasion and metastasis [20]. The expression of lnc-Ma301 in HCC tissues was lower than in adjacent normal tissue. Functional and mechanistic analyses found that lnc-Ma301 inhibited cell proliferation, migration, and EMT by targeting cytoplasmic activation/proliferation associated protein-1 (caprin-1) through the Akt/Erk1 pathway.

## Materials and methods

### Patient tissue samples

A consecutive series of 216 HCC patients (≥ 18 years) who underwent curative hepatectomy at the Guangxi Medical University Cancer Hospital (Nanning, China) between January 2014 and September 2014 were included in the study. Diagnoses of HCC was confirmed by postoperative histopathology. Patients who received neoadjuvant therapies were excluded. Fresh HCC tissues and corresponding adjacent normal tissues were obtained from our tumor tissue bank [12, 21]. All procedures were performed according to the Declaration of Helsinki (2013 version). The study was approved by the Ethics Committee of Guangxi Medical University Cancer Hospital. The requirement for written informed consent was waived because of the retrospective nature of the study.

### Cell cultures and M1/M2 phenotype identification

U937 cells (ATCC, CRL-1593.2, USA) were plated in 24-well plates at a density of 2 × 10^6^ cells per well and cultured with fresh RPMI-1640 medium (A1049101, Thermo Fisher Scientific, USA) supplemented with 5% fetal bovine serum (0024012DJ, Thermo Fisher Scientific, USA). To induce the M2 phenotype, the cells were stimulated with phorbol 12-myristate 13-acetate (PMA, 100 ng/mL, P1585, Sigma-Aldrich, USA) for 24 h, followed by the addition of interleukin (IL)-4 (20 ng/mL, PHC0044, Thermo Fisher Scientific, USA) and IL-13 (20 ng/mL, PHC0135, Thermo Fisher Scientific, USA) for 12 h. The switch from the M2 to the M1 phenotype was obtained by pretreatment with PMA (100 ng/mL) for 24 h, followed by incubation with lipopolysaccharide (LPS; 100 ng/mL, L2630, Sigma-Aldrich, USA) and interferon (IFN)-γ (20 ng/mL, GF305, Sigma-Aldrich, USA). Cellular phenotypes were identified by analyzing the expression of transforming growth factor (TGF)-β, arginase 1 (Arg-1), and tumor necrosis factor α (TNFα) by quantitative PCR (qPCR) analysis. Western blotting analysis was used to quantify the expression of Arg-1 and inducible nitric oxide synthase (iNOS) protein. An enzyme-linked immunosorbent assay (ELISA) was used to assay secreted IFN-γ, TGF-β, and IL-10. Duplicate samples of the identified M1 and M2 cells were stored at − 80 °C for subsequent analysis.

### Microarray analysis

Total RNA was isolated from M1 and M2 macrophages using TRIzol® reagent according to the manufacturer’s instructions. The mRNA was further purified using an mRNA-ONLY™ Eukaryotic mRNA Isolation kit (Epicentre, Madison, WI, USA) according to the manufacturer’s instructions. RNA quantity was examined using a NanoDrop® ND-1000 (Thermo Scientific, Scotts Valley, CA, USA), and RNA integrity was evaluated by standard denaturing agarose gel electrophoresis. Sample labeling and Agilent array hybridizations were conducted according to the manufacturer’s instructions for one-color microarray-based gene expression analysis (Agilent Technology, Santa Clara, CA, USA). Each sample was randomly primed using an Arraystar Flash RNA Labeling Kit (Arraystar, Rockville, MD, USA) and transcribed as fluorescently labeled complementary RNA (cRNA), which was then purified using a RNeasy® Mini Kit (Qiagen, Hilden, Germany). Each labeled cRNA sample (1 μg) was fragmented by adding 5 µL of blocking agent (Qiagen, Hilden, Germany) and 1 µL of fragmentation buffer, followed by heating at 60 °C for 30 min and then diluting the samples with 25 µL of GE hybridization buffer. Next, 50 mL of hybridization solution were added to the gasket slide and then lncRNA microarray slides were assembled. All slides were then incubated at 65 °C for 17 h in an Agilent hybridization oven. Finally, the hybridized arrays were washed, fixed, and scanned using the Agilent DNA Microarray Scanner (G2565BA; Agilent). lncRNA expression profiles were analyzed using the SBC Human (4 × 180 K) lncRNA Microarray version 3.0 (Arraystar LncRNA microarray, USA). The obtained lncRNA expression data were then analyzed using GeneSpring software (Agilent Technologies, USA). Fold change was used to identify lncRNAs and mRNAs differentially expressed between M1 and M2 cells. Multiple tests were used to calculate adjusted *P* values (*q* value).

### Cell lines, cell migration, invasion, and proliferation assays

SMMC-7721, QGY-7703, HepG2, HL-7702, and Huh-7 cell lines were used to detect the expression of lnc-Ma301. Cell migration and invasion assays using SMMC7721 cells were performed in 24-well chambers with 8-µm transwell inserts (Falcon). The cells were seeded in serum-free Dulbecco’s Modified Eagle medium (DMEM; GIBCO, USA) added to the top chamber. For invasion assays, the chambers were precoated with Matrigel (catalogue no.354230, BD) that had been diluted with DMEM at a 1:3 ratio, and then left to solidify for 30 min at 37 °C. SMMC7721 cells (2 × 10^4^) were suspended in 100 µL DMEM and 0.2% BSA, then added to the upper chamber. The cells were left to migrate for 4 h or invade for 24 h at 37 °C. Quantification was performed by counting the mean number of cells in five random fields per chamber using a light microscopy (TS100-F, Nikon, Japan). Cell proliferation assays were performed using the CCK8 method. After transfection, SMMC7721 cells (100 μL, 1 × 10^4^ cells) were seeded in 96-well plates and cultured for 48 h.

### ELISA

IL-12 and IL-10 levels in supernatants of M1 and M2 cultures were quantified using an ELISA kit (Invitrogen, UK) according to the manufacturer’s instructions. Briefly, plates were coated with capture anti-IL-10 (MA5-23796, Invitrogen, USA) and anti-IL-12 (PA5-18741, Invitrogen, USA) antibodies in 50 µL/well of carbonate buffer and incubated overnight at 4 °C. A standard curve was prepared using two-fold serial dilutions of the initial concentrations of IL-10 (10 ng/mL) or IL-12 (50 ng/mg) in 1% phosphate-buffered saline (PBS)-BSA in a total volume of 50 µL/well. The samples were then incubated at room temperature with biotinylated antibodies at final concentrations of 2 µg/mL for anti-IL-10 antibody or 1 µg/mL for anti-IL-12 antibody followed by addition of AMDEX streptavidin-peroxidase (Sigma, UK). The reaction was stopped by adding 20 µL of 1 mM H_2_SO_4_ once the standard wells showed an intense blue color, and the plates were read at 450 nm using a spectrophotometer (V-5000H, Shanghai Metash Instruments Co., Ltd).

### Western blot analysis

M1 and M2 macrophages were harvested, washed, re-suspended in 100 μL of lysis buffer [20 mmol/L HEPES (pH 7.4), 0.5% Nonidet P-40 (v/v), 1 mmol/L EDTA, 2 mmol/L dithiothreitol, 1 mmol/L PMSF, 100 mmol/L NaCl, 2 mmol/L Na_3_VO_4_], mixed with NuPAGE LDS buffer (Life Technologies, Carlsbad, CA, USA) and resolved by SDS-PAGE on a NuPAGE 4–12% gel (Life Technologies). The samples were then transferred onto a Trans-Blot nitrocellulose membrane (BioRad, Hercules, CA, USA) for western blotting. Membranes were washed and incubated for 1 h in 5% skimmed milk in TBS and then incubated with primary anti-Arg-1 (1:1000), anti-iNOS (1:500) or anti-β-actin (1:200; Abcam, UK) overnight at 4 °C. Next, samples were incubated with goat-anti-rabbit horseradish peroxidase-conjugated IgG (1:1000) for 1 h and visualized using an enhanced chemiluminescence kit (Amersham Pharmacia Biotech, Piscataway, NJ, USA) according to the manufacturer’s protocol. Other antibodies included E-cadherin (catalog no. 20874-1-AP, Proteintech, China) (1:5000), vimentin (10366-1-AP, Proteintech) (1:2000), matrix metalloproteinase 9 (MMP9) (10375-2-AP, Proteintech) (1:1000), slug (ab27568, Abcam, UK) (1:1000), Ki67 (27309-1-AP, Proteintech) (1:1000), anti-AKT1 (ab179463, Abcam) (1:1000), anti-ERK-3 (ab53277, Abcam) (1:1000), caprin-1 (ab241071, Abcam) (1:1000), and GAPDH (ab9484, Abcam) (1:5000) were used to detect relevant proteins.

### Quantitative real-time PCR (qRT-PCR)

qRT-PCR was performed using a SYBR Premix Ex Taq kit (RR820A, Takara, Japan) according to the manufacturer’s instructions. The primer sequences are listed in Additional file 1: Table S1. Data were collected and analyzed using a LightCycler 480 instrument (Roche, USA) with the 2ΔΔCt method.

### Cell lines over- and underexpressing lnc-Ma301 and caprin-1

The construct pLCDH-lnc-Ma301 (pLCDH is abbreviated as pL in context) was prepared by inserting the lnc-Ma301 sequence (primer sequences in Additional file 1: Table S1) into the polylinker region of pL-CMV-MCS-EF1-GFP + Puro (CD513B-1; Geneseed Biotech, Guangzhou, China) using XbaI and EcoRI restriction enzymes. The full-length sequence of human EST031.1 was obtained by rapid amplification of cDNA ends using RACE and extended. The plasmid was sequenced to confirm that the target sequence had been inserted correctly. Endotoxin-free plasmid was extracted using the E.Z.N.A. ® Endo-free Plasmid Mini Kit I (Omega) and stored at − 20 °C.

The plasmid was packaged into lentivirus using 293T cells (ATCC CRL-11268). Cells stably expressing lnc-Ma301 were harvested and analyzed by qRT-PCR, western blotting, and cell functional assays. As a control to verify the effects of lnc-Ma301, it was targeted specifically using a small interfering RNA (siRNA), siR-lnc-Ma301 (5′-CCAGUGUGAGUGAUGUUUATT-3′). As a negative control, the scrambled sequence siR-NC (5′-UUCUCCGAACGUGUCACGUTT-3′) was prepared.

We performed analogous steps to under- and overexpress caprin-1. The construct pcDNA3.1-caprin-1 (pcDNA3.1 is abbreviated as pC in context) was prepared by inserting the caprin-1 sequence (forward primer, 5′-CTGCACAGCCTATGAATCCAAC-3′; reverse primer, 5′-TTGAGATGCTGTGTACCCCTC-3′) (2148 bp) between the BamHI (GGATCC) and XhoI restriction enzymes in pcDNA3.1. The plasmid was amplified in Top 10 cells. The siRNA siR-caprin-1 (5′-GGAGCAGCUUAUGAGAGAATT-3′) was prepared, as well as the negative control siR-NC (5′-UUCUCCGAACGUGUCACGUTT-3′).

### RNA pull-down assay

The MEGAscript™ T7 High Yield Transcription kit (Invitrogen, USA) was used to transcribe biotin-labelled RNAs in vitro. Bio-16-UTP (10 mM, Ambion) was used for transcription. After adding 2 µL of Dnase I, the Eppendorf tube was incubated at 37 °C for 15 min to remove the DNA, then 2 µL of 0.2 M EDTA (pH 8.0) was added. In order to allow the RNA to form secondary structure, 1 µg of biotinylated RNA in RNA structure buffer was heated at 95 °C for 2 min, put on ice for 3 min, then left at room temperature for 30 min. Magnetic beads (Invitrogen, USA) were used to bind and enrich the RNAs. Folded RNA was then mixed with cytoplasmic extract from liver cancer cells in 500 µL RIP wash buffer. The magnetic beads were re-suspended in 50 µL RIP wash buffer, then the suspension was added to Dynabeads M-280 Streptavidin (60210, Invitrogen) and incubated at 4 °C. The suspension was centrifuged for 1 min, and the supernatant was discarded. Magnetic beads were washed briefly with RIP wash buffer for six times and boiled in SDS buffer. The retrieved proteins were detected by western blot and mass spectrometry. RNA probes were as follows: lnc-Ma301 sense: taatacgactcactatagggGGAGAGTTTGGGTCACAGGAGC, lnc-Ma301 antisense: CCTACTTGTTTTTTTTATTTTGG.

### RNA immunoprecipitation

RNA immunoprecipitation was performed using a Magna RIP RNA-Binding Protein Immunoprecipitation kit (17–700, Millipore) according to the manufacturer’s instructions. Antibody against caprin-1 (ab241071, Abcam) was used. The proteins enriched by the probe were collected, digested, and identified by FT-ICR-MS (solariX XR ESI, Bruker, USA) followed by the confirmation based on the protein data searching.

### RACE assay

Total RNA was isolated using TRIzol Plus RNA Purification Kit (Invitrogen), according to the manufacturer’s instructions. RACE was conducted using the Smart RACE cDNA Amplification Kit (Cat. No. 634923, Clontech) according to the manufacturer’s instructions. The pEASY-Blunt Simple Cloning Kit (Catalogno. CB111-01, TransGen Biotech, China) was used to clone the amplified lnc-Ma301. All selected clones were sequenced and identified. All primers used in the study are listed in Additional file 1: Table S1.

### Fluorescent in situ hybridization (FISH)

HepG2 cells were allowed to attach onto the slides, washed with PBS and fixed in 4% paraformaldehyde. All slides were treated with protease reagent, incubated with prehybridization buffer at 42 °C for 4 h and hybridized with digoxin-labeled probe overnight at 42 °C. The slides were incubated with biotin-conjugated anti-digoxin antibody (anti-digoxin-FITC) at 37 °C for 1 h and washed three times. DAPI was used to stain the cell nucleus. The images were captured using a confocal microscope (TCS SP2 AOBS). The probe sequence is listed in Additional file 1: Table S1.

### Lung metastasis in mice

Six-week-old male athymic nude mice were purchased from the Experimental Animal Center of Guangxi Medical University (Nanning, China) and housed under standard conditions at the animal care facility of the same center. Animal procedures were approved by the Guangxi Medical University Animal Care and Use Committee, and they complied with all relevant ethical regulations regarding animal research. SMMC-7721 HCC cells transfected with pL or pL-lnc-Ma301 were suspended in 200 μL PBS and injected in the tail vein of the mice (n = 5 per group, one for metastasis detection). The overexpression of lnc-Ma301 was confirmed by qRT-PCR before injection. Mice euthanized were inhalation of CO_2_ for 5 min. The CO_2_ exposure used a gradual fill method with a displacement rate about 50% of the chamber volume/min.

All mice were sacrificed 8 weeks later. Photographs were taken to assess lung metastasis, and lung sections were stained using hematoxylin and eosin to assess lung colonization. Levels of E-cadherin, MMP9, and Ki67 in lung tissues were quantified by qRT-PCR and validated by immunohistochemistry and western blotting. Each experiment was performed in triplicate and performed multiple times.

### Statistical analysis

Statistical analyses were performed using GraphPad Prism 5.01 or SPSS21.0. The Kaplan–Meier method was used to calculate the overall survival, and the significance of survival differences was determined by log-rank tests. Data from three independent experiments were presented as mean ± standard deviation (SD). Differences were evaluated for significance using two-tailed Student’s *t* test. *P* < 0.05 was considered statistically significant.

## Results

### Microarray analysis of lncRNA and mRNA during polarization from M2 to M1 cells

**Fig. 1:**
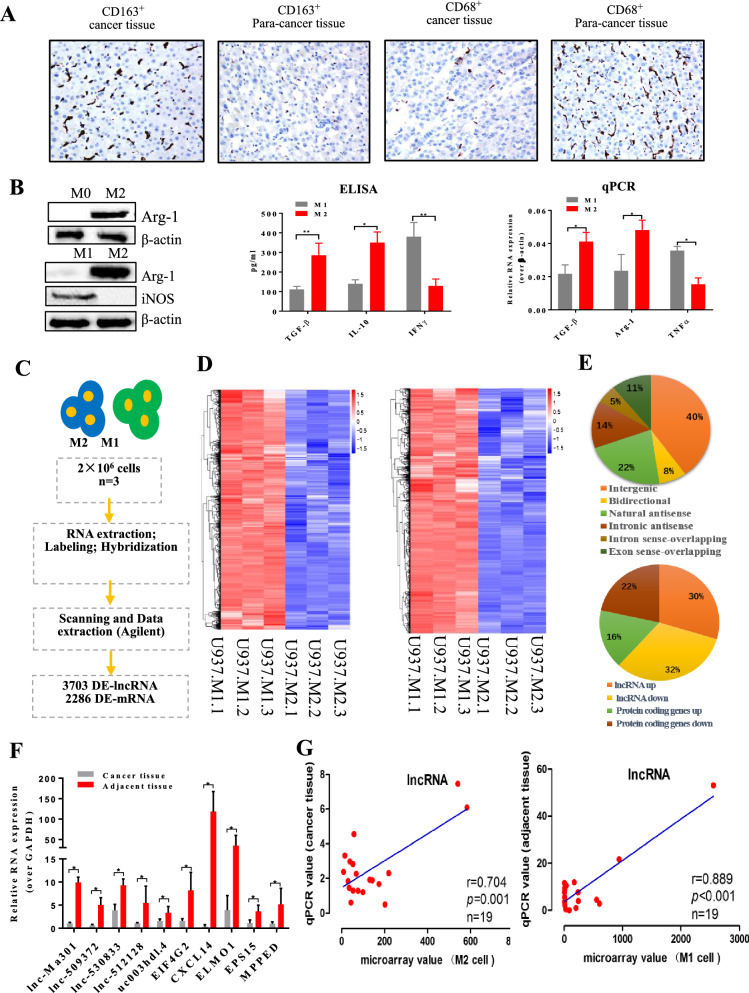
Microarray analysis of long non-coding RNAs (lncRNAs) and mRNAs during polarization from M2 to M1 macrophages. **A** CD68 and CD163 immunohistochemical staining in hepatocellular carcinoma (HCC) tissues and corresponding adjacent normal tissues (n = 30). The images represent the distributions of M1 and M2 cells inHCC tissues and adjacent normal tissues. Pictures were taken at 200× magnification. **B** M2 and M1 phenotype identification. U937 cells gave rise to M2 phenotype macrophages after stimulation with PMA, IL-4, and IL-13, which were polarized into M1 phenotype macrophages by LPS and IFN-γ stimulation. Western blotting, ELISA and qRT-PCR were used to identify the M2 and M1 phenotypes. β-actin was used as a reference for Western blotting. Anti-Arg-1 (40 KD) antibody was used as a marker of the M2 phenotype and iNOS (130 KD) antibody as a marker of the M1 phenotype. TGF-β, IL-10, and IL-12 were also used as markers to identify M2 and M1 macrophages. **C** Workflow for lncRNA analysis. **D** Clustering and pairwise comparison of lncRNAs differentially expressed between M1 and M2 cells. **E** Distribution of six types of lncRNAs as well as down- and up-regulated lncRNAs and mRNAs. **F** qRT-PCR validation of the selected genes from the microarray data. **G** Pearson correlation analysis to assess relationships between microarray and qRT-PCR results

At first, immunohistochemical staining was performed based on 30 paired HCC tissues and adjacent normal tissues. We found that the expression of CD163 was significantly higher in HCC tissues than in the adjacent tissues. Conversely, the expression of CD68 was significantly lower in normal tissues than in adjacent tissues (Fig. 1A).

Stimulation of U937 cell cultures with PMA, IL4, and IL13 induced the M2 phenotype, as described before [22]. A switch from M2 to M1 phenotype was promoted by LPS and IFN-γ stimulation, as described before [23, 24]. Western blot, ELISA, and qRT-PCR were used to confirm the M2 and M1 phenotypes after stimulation of U937 cells with different activators (Fig. 1B). Anti-Arg-1 antibody was used as a marker for the M2 phenotype and anti-iNOS antibody for the M1 phenotype. ELISA was used to detect proteins secreted by U937 cells. TGF-β and IL-10 were the two main secretory proteins of M2 macrophages, while IFN-γ was the main secretory protein of M1 macrophages. qRT-PCR was used to detect transcript levels of TGF-β, Arg-1 and TNF-α. TGF-β and Arg-1 were biomarkers of M2 macrophages, while TNF-α was a biomarker of M1 macrophages.

The workflow for lncRNA analysis is described in Fig. 1C. Microarray analysis was performed to identify lncRNAs and mRNAs differentially expressed between M1 and M2 cells. Clustering and pairwise comparison of lncRNAs differentially expressed between M1 and M2 cells are shown in Fig. 1D. We uncovered 26,276 lncRNAs differentially expressed between M1 and M2 phenotypes of U937 macrophages. lncRNAs were classified as intergenic, bidirectional, natural antisense, intronic antisense, intron sense-overlapping, or exon sense-overlapping. The distribution of these six types of lncRNAs as well as the distribution of down- and up-regulated lncRNAs and genes are shown in Fig. 1E. Nineteen lncRNAs were selected to verify the relationship between microarray data and qRT-PCR examination (Fig. 1G), and agreement between the two analyses was confirmed using Pearson correlation analysis. By qRT-PCR, we found that lnc-Ma301 was one of the most highly expressed lncRNAs in HCC tissues (Fig. 1F). Microarray analyses indicated that lnc-Ma301 was one of the most highly up-regulated lncRNAs in M1 compared to M2 cells.

### Expression and localization of lnc-Ma301

**Fig. 2:**
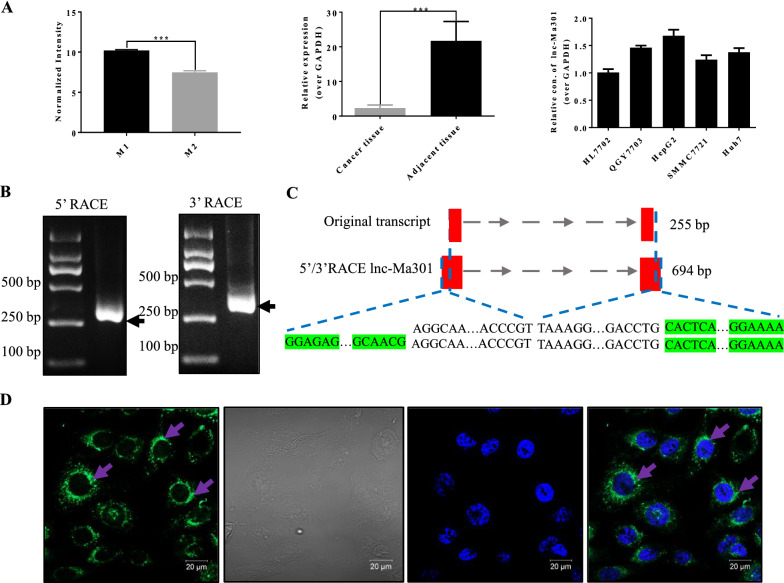
Macrophage polarization-associated lnc-Ma301 is linked to hepatocellular carcinoma (HCC). **A** qRT-PCR validation of lnc-Ma301 expression in M1/M2 macrophages and HCC tissues (n = 14) and cell lines. **B** Agarose gel electrophoresis of the 5’ and 3’ RACE amplification products of lnc-Ma301. **C** Schematic presentation of full-length lnc-Ma301 showing the extended regions identified by RACE (green). The full-length of lnc-Ma301 was 694 base pairs (bp), amplified from the original 255 bp. **D** Representative images of RNA FISH showing nuclear localization of lnc-Ma301 (green) in HCC cell line HepG2 cells. Nuclei were stained with DAPI (blue). Scale bar indicates 20 μm. Data are presented as mean ± standard error of the mean (SEM). *P < 0.05; ***P < 0.001

The expression of lnc-Ma301 in M1 macrophages was significantly higher than that in M2 macrophages (Fig. 2A, left). Moreover, in the 14 patients analyzed, the expression of lnc-Ma301 in adjacent tissue was also significantly higher than in HCC tissues (Fig. 2A, middle). Lnc-Ma301 was expressed in all cell lines including HL-7702, QGY-7703, HepG2, SMMC-7721, and Huh-7 (Fig. 2A, right), with higher expression levels in HepG2 cell line.

qRT-PCR and agarose gel electrophoresis were used to detect the 5’ and 3’ RACE amplification products of lnc-Ma301 (Fig. 2B). The full-length lnc-Ma301 was 694 base pairs (bp), amplified from the original 255 bp (Fig. 2C). FISH against lnc-Ma301 in HepG2 cell lines revealed that the lncRNA was expressed mainly around the nucleus (Fig. 2D).

### lnc-Ma301 presents low expression in HCC tissues and is associated with prognosis

**Fig. 3:**
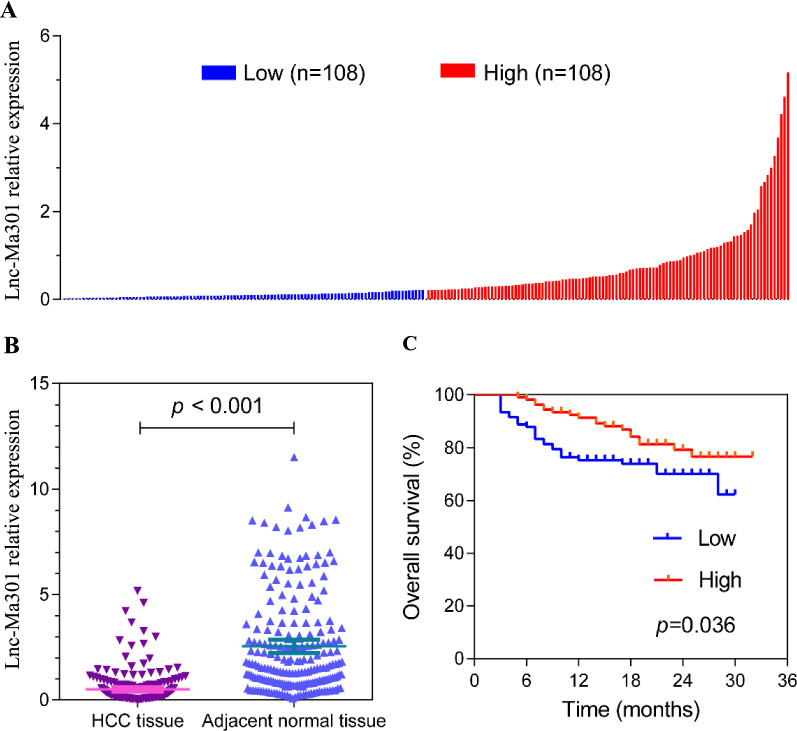
Lnc-Ma301 expression in 216paired hepatocellular carcinoma (HCC) and adjacent normal tissues. **A** lnc-Ma301 expression in HCC patients (the threshold was established according to the median value of relative lnc-Ma301 expression in HCC tissues). Each experiment was performed in triplicate. **B** Relative expression level of lnc-Ma301 in HCC tissues and adjacent normal tissues. **C** Kaplan–Meier curve of the prognostic value of lnc-Ma301 expression for HCC patients

The baseline characteristics of the 216 HCC patients are described in Table 1 and Additional file 2: Table S2. qRT-PCR was used to detect the expression of lnc-Ma301 in 216 HCC tissues and adjacent normal tissues (Fig. 3A). Expression was classified as high or low using the median value of relative lnc-Ma301 expression in HCC tissues as a threshold. The expression of lnc-Ma301 in HCC tissues was significantly lower than in adjacent normal tissues (Fig. 3B). Moreover, lower expression of lnc-Ma301 in HCC tissues was associated with lower overall survival (Fig. 3C). These results suggest that lnc-Ma301 may be a protective factor for patients with HCC and may show utility as a prognostic marker.

**Table 1:** Relationship of lncMa-301 expression and overall survival with demographic and clinical data in HCC patients

Variable	lncMa-301 expression				Overall survival analysis		
	Low (n = 108)	High (n = 108)	Odds ratio (95%CI)	*P*	No. of patients	Hazard ratio (95%CI)	*P*
Sex							
Female	13	16	0.79 (0.36–1.73)	0.549	29	Ref.	0.184
Male	95	92	Ref.		187	2.00 (0.72–5.57)	
Age, year							
< 60	84	90	0.70 (0.36–1.38)	0.302	174	Ref.	0.144
≥ 60	24	18	Ref.		42	0.53 (0.23–1.24)	
Size, cm							
< 5	29	35	0.77 (0.43–1.38)	0.371	64	Ref.	**0.016**
≥ 5	79	73	Ref.		152	2.67 (1.20–5.95)	
Number of tumors							
< 3	97	99	0.80 (0.32–20.2)	0.639	196	Ref.	**0.023**
≥ 3	11	9	Ref.		20	2.42 (1.13–5.17)	
Tumor capsule							
Complete	59	65	0.80 (0.46–1.37)	0.409	124	Ref.	**0.015**
Incomplete/absent	49	43	Ref.		92	2.03 (1.15–3.58)	
Lymph node metastasis							
No	99	104	0.42 (0.13–1.42)	0.153	203	Ref.	**0.006**
Yes	9	4	Ref.		13	3.06 (1.37–6.81)	
Macrovascular invasion							
No	66	69	0.89 (0.51–1.54)	0.673	135	Ref.	** < 0.001**
Yes	42	39	Ref.		81	2.92 (1.65–5.16)	
Microvascular invasion							
No	42	45	0.89 (0.52–1.54)	0.677	87	Ref.	** < 0.001**
Yes	66	63	Ref.		129	4.04 (1.89–8.62)	
AFP, ng/mL							
< 400	53	55	0.93 (0.55–1.58)	0.785	108	Ref.	0.181
≥ 400	55	53	Ref.		108	1.47 (0.84–2.59)	
BCLC stage							
0/A	47	51	0.86 (0.50–1.47)	0.585	98	Ref.	**0.001**
B/C	61	57	Ref.		118	2.94 (1.53–5.65)	
EST031.1 expression							
Low	108	0		NA	108	Ref.	**0.036**
High	0	108			108	0.54 (0.30–0.96)	

Bold values indicate *P* value less than 0.05

*AFP* alpha fetoprotein, *BCLC* Bacelona Clinic Liver Cancer, *CI* confidence interval, *HCC* hepatocellular carcinoma, *Ref.* reference

### lnc-Ma301 inhibits proliferation and migration of HCC cells as well as the EMT in vitro

**Fig. 4:**
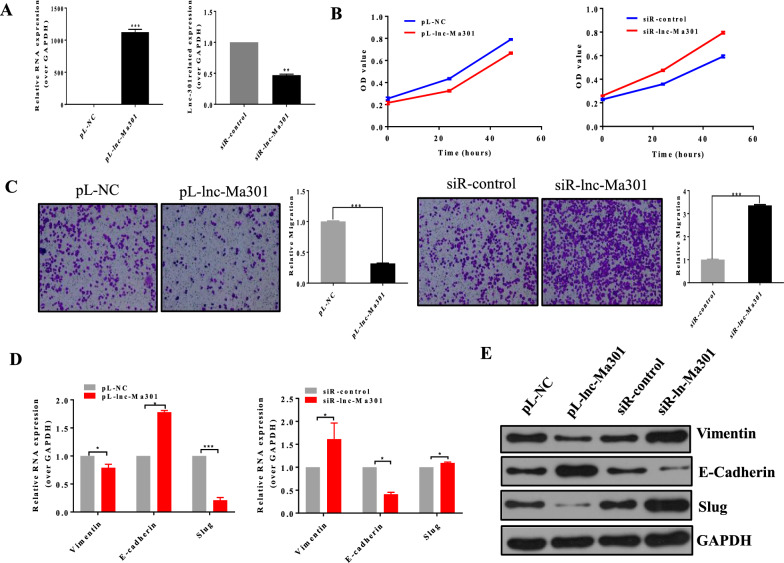
Lnc-Ma301 inhibits proliferation and migration of HCC cells in vitro. **A** Overexpression and silencing of lnc-Ma301 in SMMC7721 cells. **B** CCK8 assay evaluating the effect of lnc-Ma301 on cell proliferation. **C** Transwell assay to determine the effect of lnc-Ma301 on cell migration. **D** Analysis of the expression of EMT-associated genes vimentin, E-cadherin, and slug by qRT-PCR. **E** Levels of EMT-associated proteins vimentin, E-cadherin, and slug by western blotting. Data are presented as mean ± standard error of the mean (SEM). *P < 0.05; ***P < 0.001

Overexpression and silencing of lnc-Ma301 was verified in SMMC7721 cells (Fig. 4A). Overexpression of lnc-Ma301 suppressed the proliferation of SMMC7721 cells, while its silencing promoted cell proliferation (Fig. 4B). Overexpression of lnc-Ma301 also suppressed the migration of SMMC7721 cells in transwell assays, while its silencing promoted migration (Fig. 4C).

The expression of EMT-associated genes vimentin, E-cadherin, and slug was analyzed by qRT-PCR (Fig. 4D) and western blotting (Fig. 4E). E-cadherin promotes progression through the EMT, while vimentin and slug inhibit the transition [25]. The expression of E-cadherin in pL-lnc-Ma301 cells was significantly higher than that of the empty control, while the expression of vimentin or slug in pL-lnc-Ma301 cells was significantly lower than that of the empty control. The opposite effect was observed in siR-lnc-Ma301 and siR-control cells. Together, these results confirmed that lnc-Ma301 inhibits HCC cell proliferation and migration as well as the EMT in vitro.

### lnc-Ma301 inhibits lung metastasis in vivo

**Fig. 5:**
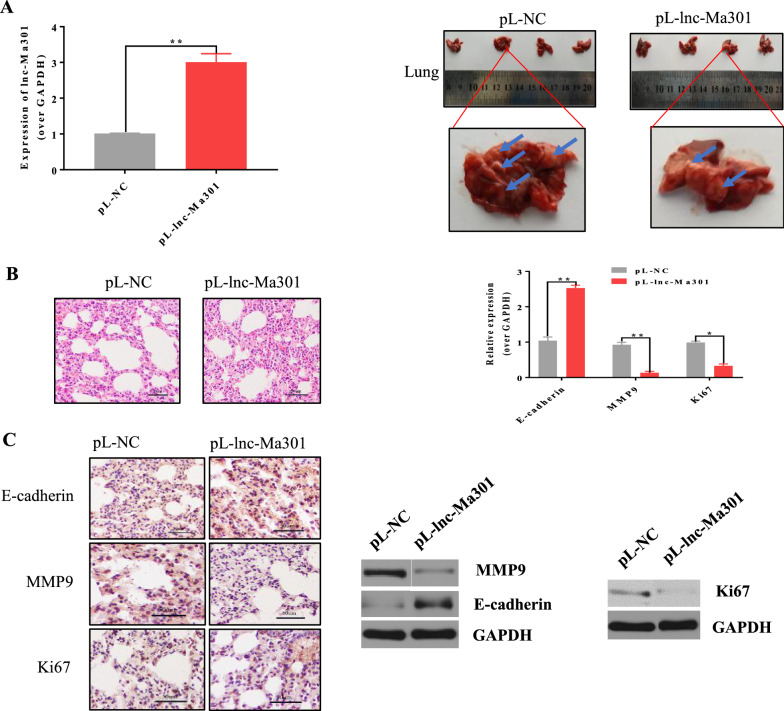
Lnc-Ma301 inhibits the lung metastasis ability of HCC cells in vivo. **A** A lung metastasis model was established by injection of SMMC-7721 HCC cells into the tail vein of nude mice, and lung metastasis was investigated. Overexpression of lnc-Ma301 was confirmed by qRT-PCR before injection. Photographs of lung metastasis were taken (blue arrow indicates tumor nodule). Amagnified lung in each group is shownas an example. **B** Histological examination of lung colonization. Magnification, × 200. Expression of E-cadherin, MMP9, and Ki67 in the lung tissues was quantified by qRT-PCR. **C** Immunohistochemistry was used to validate the protein levels of E-cadherin, MMP9, and Ki67 in each group. Western blotting was also used to confirm the protein levels of E-cadherin, MMP9, and Ki67. Experiments were performed in triplicate in each experiment. *P < 0.05

In order to investigate the function of lnc-Ma301 in lung metastasis in vivo, a lung metastasis model was established in which nude mice were injected with pL-lnc-Ma301-SMMC-7721 or pL-SMMC-7721 HCC cells through the tail vein. qRT-PCR analysis revealed that the expression of lnc-Ma301 in lung tissues of pL-lnc-Ma301-SMMC-7721 mice was significantly higher than in the pL-SMMC-7721 group (Fig. 5A, left). Moreover, the number of lung metastases in the pL-lnc-Ma301-SMMC-7721 mice was significantly lower than that in the pL-SMMC-7721 group (Fig. 5A, right).

Next, we used hematoxylin–eosin staining to investigate lung colonization. The degree of tumor cell infiltration was significantly lower in the pL-lnc-Ma301-SMMC-7721 group than in the control group (Fig. 5B, left). This suggests that lnc-Ma301 inhibited neoplasia.

MMP9 and E-cadherin are involved in tumor invasion and metastasis [26], while Ki67 is a well-known proliferation marker for the evaluation of cell proliferation [27]. Therefore, the expression of E-cadherin, MMP9, and Ki67 in normal lung tissues and metastatic tissues were quantified by qRT-PCR and validated by immunohistochemistry and western blotting. The expression of E-cadherin in the pL-lnc-Ma301-SMMC-7721 group was significantly higher than in the control group (Fig. 5B, right). This finding was confirmed by immunohistochemistry (Fig. 5C, left) and western blotting (Fig. 5C, right). The opposite findings were observed for MMP9 and Ki67 (Fig. 5B, C). Therefore, our data suggest that lnc-Ma301 inhibits HCC cell line lung metastasis by inhibiting cell proliferation, tumor invasion and metastatic potential.

### Caprin-1 interacts with lnc-Ma301

**Fig. 6:**
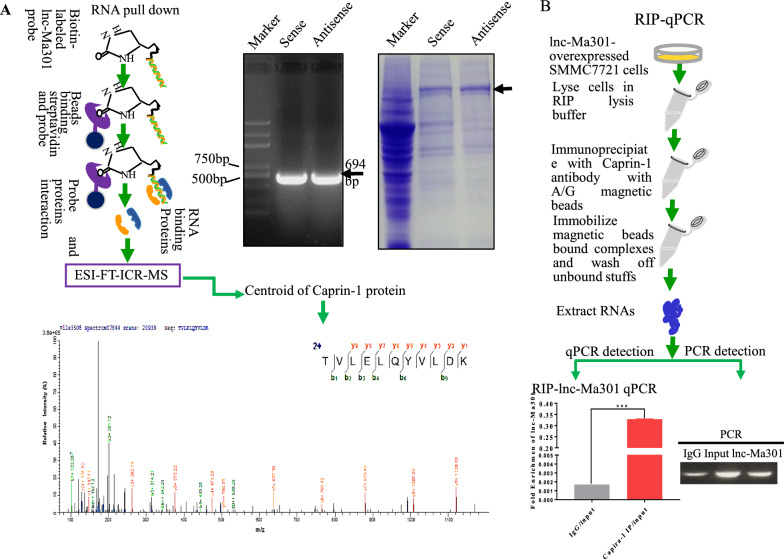
Caprin-1 protein interacts with lnc-Ma301. **A** Scheme of the RNA pull-down assay to identify proteins interacting with lnc-Ma301. **a** Both sense and antisense probes of lnc-Ma301 were verified by electrophoresis. **b** The pulled-down proteins by both sense and antisense probes of lnc-Ma-301 were analyzed by ESI-FT-ICR-MS. Differentially expressed proteins were those pulled down by the sense probe but not the antisense probe. **c,** A specific peptide was used to identify caprin-1 protein. **B** Caprin-1 protein was probed further as a potential interactor with lnc-Ma301, and its antibody was used to perform the RIP-qPCR to confirm their interaction

Predicting the interaction between lncRNAs and proteins is a useful way to explore the functions of lncRNAs [28]. Therefore, a lnc-Ma301-RNA pull-down assay was performed to identify the interacting proteins (Fig. 6A, left). Both sense and antisense probes of lnc-Ma301 were evaluated in pull-downs (Fig. 6A, middle), and the associated proteins were analyzed by ESI-FT-ICR-MS (Fig. 6A, right). The proteins interacting with lnc-Ma301 were defined as proteins pulled down by the sense but not the antisense probe.

Among the interactors, caprin-1 is involved in tumor proliferation, migration,and invasion [29] as well as prognosis in HCC [30]. The overexpression of caprin-1 may contribute to the growth and invasion of several types of tumor cells [31]. Therefore we focused on this protein in subsequent analyses. The caprin-1 specific peptide identified by ESI-FT-ICR-MS was confirmed as the interacting protein of lnc-Ma301 (Fig. 6A). Next, an RIP assay was performed to confirm whether caprin-1 interacts with lnc-Ma301 (Fig. 6B, left). The antibody of caprin-1 was used to perform the RIP-qPCR to confirm the interaction between caprin-1 and lnc-Ma301 (Fig. 6B, right). Finally, the result of the qPCR on the pulled-down RNA showed that the mRNA encodes lnc-Ma301 (Fig. 6B). Together, these data suggest that caprin-1 protein interacts with lnc-Ma301.

### lnc-Ma301 inhibits cell proliferation, migration, and EMT in vitro by interacting with caprin-1

**Fig. 7:**
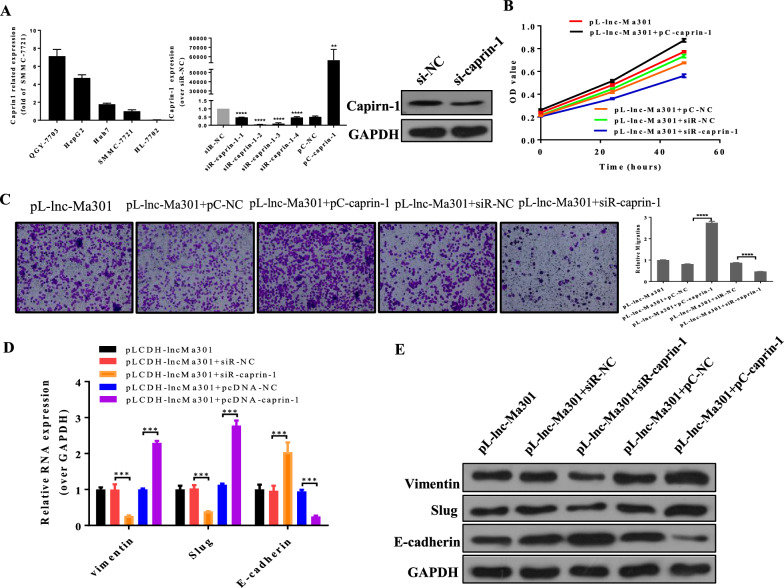
Lnc-Ma301 inhibits the proliferation and migration of HCC cells via interaction with caprin-1 in vitro. **A** The expression of caprin-1 in different cell lines (left). Confirmation of the effects of caprin-1 silencing (si) (middle) and overexpression (OE) (right). **B** The CCK8 assay was used to confirm the effect of lnc-Ma301 and caprin-1 interaction on cell proliferation. **C** A transwell assay was used to determine the effect of lnc-Ma301 and caprin-1 interaction on cell migration. **D** The expression of EMT-associated genes vimentin, E-cadherin, and slug were analyzed by qRT-PCR. **E** Levels of EMT-associated proteins vimentin, E-cadherin, and slug were analyzed by western blotting. Data are presented as mean ± standard error of the mean (SEM). *P < 0.05

After examination of caprin-1 level in HCC cell and normal liver cell lines (Fig. 7A), we silenced caprin-1 by four designed siRNAs in SMMC7721 cell lines, the siRNA caprin-1–2 resulted in the most significant downregulation of caprin-1, and it also decreased the protein level of caprin-1, so it was selected for subsequent experiments (Fig. 7A). The simultaneous overexpression of lnc-Ma301 and caprin-1 promoted cell proliferation (Fig. 7B) and migration (Fig. 7C), while the overexpression of lnc-Ma301 together with the silencing of caprin-1 resulted in decreased cell proliferation (Fig. 7B, C).

Next, the expression of EMT-associated genes E-cadherin, vimentin, and slug were analyzed by qRT-PCR and protein levels were confirmed by western blotting. In cells simultaneously overexpressing lnc-Ma301 and caprin-1, expression of E-cadherin was increased, while expression of vimentin and slug was decreased (Fig. 7D, E). Our results suggest that lnc-Ma301 inhibits cell proliferation and migration as well as the EMT in vitro by interacting with caprin-1.

### lnc-Ma301 interacts with caprin-1 to inhibit HCC metastasis and EMT via the Akt/Erk1 pathway

**Fig. 8:**
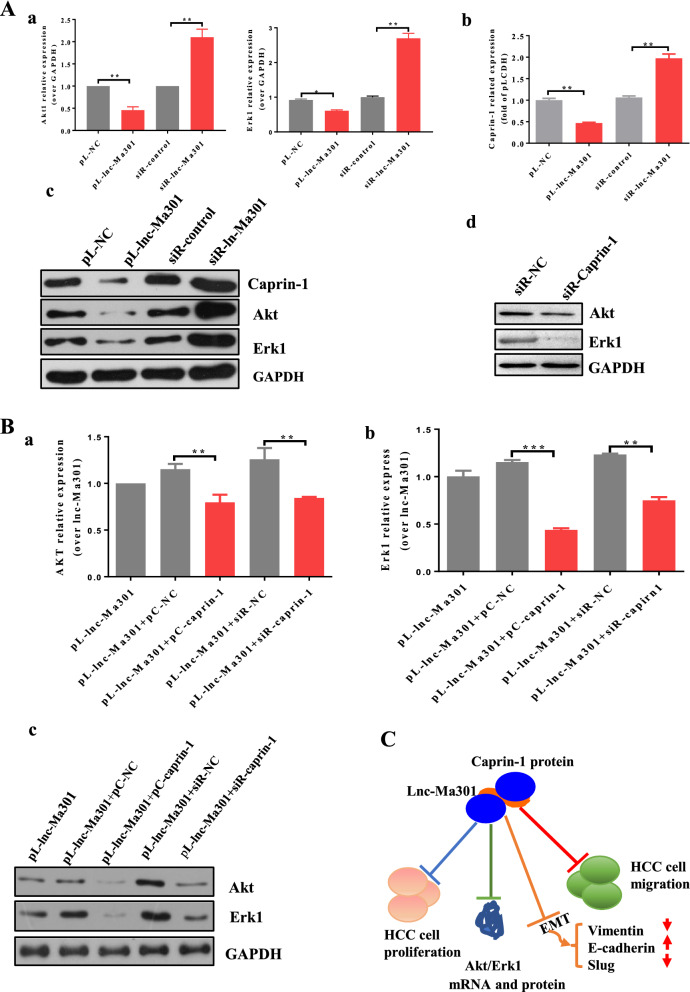
Potential mechanism for how lnc-Ma301 regulates the proliferation and migration of HCC cells. **A**, **a** Akt, Erk1, and caprin-1 expression at mRNA level when lnc-Ma301 was overexpressed or silenced in SMMC7721 cells. **b** Akt, Erk1, and caprin-1 protein levels when lnc-Ma301 was overexpressed or silenced in SMMC7721 cells. **c** Akt and Erk1 protein levels when caprin-1 was silenced in SMMC7721 cells. **B** Akt and Erk1 mRNA and protein levels when lnc-Ma301 interacted with caprin-1 in SMMC7721 cells. **C** Schematic diagram of the potential mechanism of action of lnc-Ma301 on the proliferation and migration of HCC cells. lnc-Ma301 interacts with caprin-1 to inhibit HCC metastasis and EMT via the Akt/Erk1 pathway. Data are presented as mean ± standard error of the mean (SEM). *P < 0.05

Upregulation of caprin-1 promotes lung metastasis from osteosarcoma tumor in mice by activating the Akt and Erk1 pathways [32]. We wondered whether the same might be true in HCC and whether lnc-Ma301 may be involved. First, the expression of Akt, Erk1, and caprin-1 were investigated by qRT-PCR and western blotting in SMMC7721 cell lines in which lnc-Ma301 was silenced or overexpressed. Our results showed that lnc-Ma301 inhibits the expression of Akt, Erk1, and caprin-1 (Fig. 8A, panels a, b).

Next, the expression of Akt and Erk1 were investigated in caprin-1-silenced SMMC7721 cell lines using western blotting. Silencing caprin-1 inhibited the expression of Akt and Erk1 (Fig. 8A, panel c). Similarly, Akt and Erk1 were down-regulated when both lnc-Ma301 and caprin-1 were overexpressed, or when lnc-Ma301 was overexpressed but caprin-1 was underexpressed (Fig. 8B). These results suggest that lnc-Ma301 interacts with caprin-1 to inhibit HCC metastasis and EMT via the Akt/Erk1 pathway.

## Discussion

In this study, we uncovered 26,276 lncRNAs differentially expressed between M1 and M2 phenotypes of U937 macrophages. Among these, lnc-Ma301 was found as the most highly expressed lncRNA in U937 cells, especially in M1 macrophages, while it presented lower expression in HCC tissues than in adjacent normal tissues. Low lnc-Ma301 expression was associated with reduced overall survival among HCC patients after hepatic resection. We showed that overexpression of lnc-Ma301 inhibited in vitro cell proliferation and migration as well as the EMT, and it inhibited lung metastasis in vivo. These experiments provide the first evidence that lnc-Ma301 may be a critical regulator of HCC occurrence and metastasis.

How lnc-Ma301 may inhibit cell proliferation and migration as well as the EMT and lung metastasis are unknown. A large number of lncRNAs have been shown to function as master regulators for gene expression. lncRNA-mediated gene expression involves regulation of translation, protein modification, transcription, formation of RNA–protein complexes, and the regulation of cell signaling pathways [33]. To perform most of their cellular functions, lncRNAs must interact with one or more RNA-binding proteins [34]. Pull-down assays in this study found many RNA-binding proteins that interact with lnc-Ma301, including caprin-1, which participates in the regulation of genes that control the cell cycle [35]. In fact, caprin-1 may help drive tumorigenesis in several cancers [31]. The overexpression of caprin-1 may contribute to the growth and invasion of several types of tumor cells [31]. Previous studies have shown that upregulation of caprin-1 expression is associated with poor prognosis in patients with HCC [30], and that the protein regulates the proliferation and invasion of human breast cancer cells [36], as well as promotes osteosarcoma tumor growth and lung metastasis in mice by activating the Akt and Erk1 pathways [32]. Caprin-1 is needed for normal progression through the G1-S phase of the cell cycle [35, 37]. Our results suggest that the abilities of lnc-Ma301 to inhibit the cell proliferation and migration as well as the EMT require interaction with caprin-1. This appears to be the first evidence of an association between caprin-1 and the Akt/Erk1 pathway in HCC, extending the known association between the Akt/Erk1 pathway and cell proliferation [38].

Cellular signaling pathways play a key role in various cellular processes in response to intracellular or extracellular stimuli [33]. Overwhelming evidence supports the role of Akt [39] and Erk1 [40] signaling pathways in cancers. In addition, Akt and Erk1 pathways promote cell proliferation, migration, and survival in cancer [41]. We demonstrated an association linking the lnc-Ma301/caprin-1/Akt/Erk1 axis with cell proliferation and migration as well as the EMT (Fig. 8C). This is consistent with the observation that caprin-1 is necessary for normal cell cycle progression [35, 37].

Our study presents several limitations. First, the clinical usage of lnc-Ma301 in diagnoses and as a marker of prognoses for HCC requires more sample testing and longer period of observation. Second, the existence of the ceRNA mechanism of lnc-Ma301 was not involved in this study, which will be explored in subsequent studies. Finally, further studies on the function of caprin-1, the interaction protein of lnc-Ma301, are needed, which will be further studied in our future work.

## Conclusions

Our data demonstrate that lnc-Ma301 plays a critical role in the occurrence of HCC and lung metastasis, at least partially through its ability to interact with the RNA-binding protein caprin-1 and then regulate the downstream Akt/Erk1 signaling pathway. Activation of lnc-Ma301 could help prevent the occurrence of HCC and lung metastasis. Therefore, lnc-Ma301 may serve as a potential biomarker of HCC occurrence and lung metastasis.

## Supplementary Information

**Additional file 1: Table S1.** Primer sequences.

**Additional file 2: Table S2.** Survival of HCC patients with expression of lncMa-301, based on COX risk modeling.

## Data Availability

All RNA microarray data are present in the NCBI Gene Expression Omnibus (GEO) Series (GSE127981, grouped under SuperSeriesGSE128007). Material is available upon request to H.-L.L.
