# Supplementary material for: Macrophage polarization-associated lnc-Ma301 interacts with caprin-1 to inhibit hepatocellular carcinoma metastasis through the Akt/Erk1 pathway
Source: Cancer Cell Int. 2021 Aug 10;21:422. doi: 10.1186/s12935-021-02133-1 (PMC8353734; doi:10.1186/s12935-021-02133-1)
Supplement: Supplementary file 1 — Additional file 1: Table S1. Primer sequences. [file 12935_2021_2133_MOESM1_ESM.docx]

Table S1. Primer sequences.

| **Primers for caprin-1 plasmid** | |
| --- | --- |
| caprin-1-F | 5'- CTGCACAGCCTATGAATCCAAC -3' |
| caprin-1-R | 5'- TTGAGATGCTGTGTACCCCTC -3' |
| **Sequences for siRNAs and shRNAs** | |
| lnc-Ma301-F1 | 5'-TTACAGAGAGGGAGGGACCAG-3' |
| lnc-Ma301-R1 | 5'-GCTTCCCTCTTGCCTCGTT-3' |
| si-Ma301-1 | 5'-GCAGGUGUCUGUCACCUUATT-3' |
| si-Ma 301-2 | 5'-CCAGUGUGAGUGAUGUUUATT-3' |
| si-Ma 301-3 | 5'-CCAGCAGAAGCAGCGUAAATT-3' |
| si-NC | 5'-UUCUCCGAACGUGUCACGUTT-3' |
| siR-caprin1-1 | 5'-CCAGGAAGUCACAAAUAATT-3' |
| siR-caprin-1-2 | 5'-GGAGCAGCUUAUGAGAGAATT-3' |
| siR-caprin-1-3 | 5'-GGUUUGAAUGGAGUGCCAATT-3' |
| siR-caprin-1-4 | 5'-GCAAUCAGCCCUAUUACAATT-3' |
| siR-NC | 5'-UUCUCCGAACGUGUCACGUTT-3' |
| **Primer sequences for qRT-PCR** | |
| lnc-Ma301-F1 | 5'-TTACAGAGAGGGAGGGACCAG-3' |
| lnc-Ma301-R1 | 5'-GCTTCCCTCTTGCCTCGTT-3' |
| E-cadherin-F | 5'-TTTGAAGATTGCACCGGTCG-3' |
| E-cadherin-R | 5'-CAGCGTGACTTTGGTGGAAA-3' |
| Slug-F | 5'-AGATGCATATTCGGACCCACA-3' |
| Slug-R | 5'-GCCCTCAGATTTGACCTGT-3' |
| Vimentin-F | 5'-AAGCTGCTAACTACCAAGACAC-3' |
| Vimentin-R | 5'-ATCTCAATGTCAAGGGCCAT-3' |
| β-actin human-ACTB-F | 5'-CATGTACGTTGCTATCCAGGC-3' |
| β-actin human-ACTB-R | 5'-CTCCTTAATGTCACGCACGAT-3' |
| M-actin-F | 5'-CTGAGAGGGAAATCGTGCGT-3' |
| M-actin-R | 5'-CCACAGGATTCCATACCCAAGA-3' |
| M-E-cad-F | 5'-GACCGGAAGTGACTCGAAATG-3' |
| M-E-cad-R | 5'-CCCTCGTAATCGAACACCAAC-3' |
| M-MMP9-F | 5'-AAGGGTACAGCCTGTTCCTGGT-3' |
| M-MMP9-R | 5'-CTGGATGCCGTCTATGTCGTCT-3' |
| M-KI67-F | 5'-CCTTGCTGAGAACACCACAGC-3' |
| M-KI67-R | 5'-TTGGGTGACCATCTGACTTCCT-3' |
| Caprin-1-F | 5'-CTGCACAGCCTATGAATCCAAC-3' |
| Caprin-1-R | 5'-TTGAGATGCTGTGTACCCCTC-3' |
| AKT-F | 5'-CCACTGTCATCGAACGCACC-3' |
| AKT-R | 5'-TCCTGCTTCTTGAGGCCGTC-3' |
| ERK1-F | 5'-TCAACACCACCTGCGACCTT-3' |
| ERK1-R | 5'-CGTAGCCACATACTCCGTCA-3' |
| **Primer sequences for RIP** | |
| lnc-Ma301-F | 5'-TCCCGGACACCATGTTTACA-3' |
| lnc-Ma301-R | 5'-GGGGAATCCTCAGCAAAACC-3' |
| **LNA probe for FISH** | |
| lnc-Ma301 | 5’-TGTAATTGGCTTCTTTACAGAGAGGGAGGGACCAGGTTCCCAGTGTGAGTGATGTTTAGAGGTTTTCTGAGAAGCACAAGGACTGGACTTTGACAGGAAGCAACGAGGCAAGAGGGAAGCTCGAGAACAGGTTTTGCTGAGGATTCCCCCCAAGAAAAGTGCTCCCCGGGAGGCCGTTCCTGGCACCAAGACTGGCGGAGCCACCACACCCGTTAAAGGGATCCACCTGGACAGCGATCAGGCGTGAGCCCCCCGAGGGGAAGTTGAAGTGGAAGCAGACAAGCACCTCCAAGGATCACGTAGGACCAGCAGAAGCAGCGTAAAGTATTGGAACTTTGAATCTGACATCAGAAAGACCTGCACTCAGCCGGGCACGGTGGCTCACGATTGTAATCCCAGGCCGAGGCGGTGGATCACTTGAGATTCACTGATTCTAGG-3’ |
| Sense probe for lnc-Ma301 | 5'-taatacgactcactatagggGGAGAGTTTGGGTCACAGGAGC-3' |
| Antisense probe for lnc-Ma301 | 5'-CCTACTTGTTTTTTTTATTTTGG-3' |
| **RACE assay** | |
| lnc-Ma301-F | 5'-GAAGTGGAAGCAGACAAGCACCTCCAA-3' |
| lnc-Ma301-R | 5'- CGTAGGACCAGCAGAAGCAGCGTAA-3' |
| 3'-RACE CDS primer | 5'-AAGCAGTGGTATCAACGCAGAGTACTTTTTTTTTTTTTTTTTTTTTTTTTTTTTTV |
| 3'- RACE reverse transcription primer (long) | 5'-CTAATACGACTCACTATAGGGCAAGCAGTGGTATCAACGCAGAGT–3' |
| 3'-RACE reverse transcription primer (short) | 5'-CTAATACGACTCACTATAGGGC-3' |
| nested universal primer | 5'-AAGCAGTGGTATCAACGCAGAGT-3' |
