# Supplementary material for: Macrophage polarization-associated lnc-Ma301 interacts with caprin-1 to inhibit hepatocellular carcinoma metastasis through the Akt/Erk1 pathway
Source: Cancer Cell Int. 2021 Aug 10;21:422. doi: 10.1186/s12935-021-02133-1 (PMC8353734; doi:10.1186/s12935-021-02133-1)
Supplement: Supplementary file 2 — Additional file 2: Table S2. Survival of HCC patients with expression of lncMa-301, based on COX risk modeling. [file 12935_2021_2133_MOESM2_ESM.docx]

**Table S2.** Survival of HCC patients with expression of lncMa-301, based on COX risk modeling.

| **Variable** | **No. of patients** | **Median survival time, months** | **Crude hazard ratio (95% CI)** | ***P*** | **Adjusted hazard ratio (95%CI)** | ***P*** |
| --- | --- | --- | --- | --- | --- | --- |
| **Size, cm** |  |  |  |  |  |  |
| <5 | 64 | 18 | Ref. | **0.016** | Ref. | 0.705 |
| ≥5 | 152 | 17 | 2.67 (1.20-5.95) |  | 1.19 (0.49-2.92) |  |
| **Number of tumors** |  |  |  |  |  |  |
| <3 | 196 | 18 | Ref. | **0.023** | Ref. | 0.271 |
| ≥3 | 20 | 14 | 2.42 (1.13-5.17) |  | 1.55 (0.71-3.40) |  |
| **Tumor capsule** |  |  |  |  |  |  |
| Complete | 124 | 20 | Ref. | **0.015** | Ref. | 0.067 |
| Incomplete/absent | 92 | 14 | 2.03 (1.15-3.58) |  | 1.71 (0.96-3.05) |  |
| **Lymph node metastasis** |  |  |  |  |  |  |
| No | 203 | 18 | Ref. | **0.006** | Ref. | **0.034** |
| Yes | 13 | 15 | 3.06 (1.37-6.81) |  | 2.67 (1.08-6.62) |  |
| **Macrovascular invasion** |  |  |  |  |  |  |
| No | 135 | 19 | Ref. | **<0.001** | Ref. | **0.018** |
| Yes | 81 | 15 | 2.92 (1.65-5.16) |  | 2.29 (1.16-4.55) |  |
| **Microvascular invasion** |  |  |  |  |  |  |
| No | 87 | 19 | Ref. | **<0.001** | Ref. | **0.034** |
| Yes | 129 | 16 | 4.04 (1.89-8.62) |  | 2.45 (1.07-5.60) |  |
| **EST031.1 expression** |  |  |  |  |  |  |
| Low | 108 | 16 | Ref. | **0.036** | Ref. | **0.038** |
| High | 108 | 19 | 0.54 (0.30-0.96) |  | 0.54 (0.30-0.97) |  |

Abbreviations: CI, confidence interval; Ref., reference.
